# Pan‐cancer analysis of DCTN2 and its tumour‐promoting role in HCC by modulating the AKT pathway

**DOI:** 10.1111/jcmm.18450

**Published:** 2024-06-06

**Authors:** Shuning Xu, Jiyuan Xing, Ke Li, Lei Qiao, Cheng Zhang, Yulin Ren, Ying Liu

**Affiliations:** ^1^ Department of Medical Oncology The Affiliated Cancer Hospital of Zhengzhou University, Henan Cancer Hospital Zhengzhou China; ^2^ Department of Infectious Diseases The First Affiliated Hospital of Zhengzhou University Zhengzhou China

**Keywords:** AKT pathway, dynactin subunit 2, hepatocellular carcinoma, pan‐cancer

## Abstract

Dynactin subunit 2 (DCTN2) has been reported to play a role in progression of several tumours; however, the involvement of DCTN2 in potential mechanism or the tumour immune microenvironment among various cancers still remains largely unknown. Therefore, the objective of this study was to comprehensively investigate the expression status and potential function of DCTN2 in various malignancies through different database, such as The Cancer Genome Atlas, the Genotype‐Tissue Expression and Gene Expression Omnimus databases. We discovered that DCTN2 expression was high in many type of tumours tissues compared to adjacent non‐tumour ones. High DCTN2 signified poor prognosis for patients with tumours. Additionally, Gene Set Enrichment Analysis (GSEA) analysis revealed that DCTN2 was positively correlated with oncogenic pathways, including cell cycle, tumour metastasis‐related pathway, while it was negatively with anti‐tumour immune signalling pathway, such as INF‐γ response. More importantly, we elucidated the functional impact of DCTN2 on hepatocellular carcinoma (HCC) progression and its underlying mechanisms. DCTN2 expression was much higher in HCC tissues than in adjacent non‐tumour tissues. Silencing DCTN2 dramatically suppressed the proliferative and metastasis capacities of tumour cell in vitro. Mechanistically, DCTN2 exerted tumour‐promoting effects by modulating the AKT signalling pathway. DCTN2 knockdown in HCC cells inhibited AKT phosphorylation and its downstream targets as well. Rescue experiments revealed that the anti‐tumour effects of DCTN2 knockdown were partially reversed upon AKT pathway activation. Overall, DCTN2 may be a potent biomarker signifying tumour prognosis and a promising therapeutic target for tumour treatment, particularly in HCC.

## 1 INTRODUCTION

In recent years, there has been a progressive upsurge in cancer incidence, making it a principal global cause of mortality and imposing an overwhelming strain on the global healthcare system, such as the health and economic aspects.^1^ Despite continuous advancements in treatment methods, surgical resection remains the most effective treatment modality. However, most patients are already in advanced stages of the disease when being diagnosed, missing the optimal treatment window and resulting in a need for improved overall survival rates. Recently, the emergence of immunotherapy holds promise in breaking this impasse.^2^ Immune checkpoint inhibitors have demonstrated substantial efficacy against various tumours, including haematological malignancies, lung cancer, and gastric cancer.^3^, ^4^ Nevertheless, only a small fraction of patients derive tangible benefits from these innovative medicines. The magnificent contributing factor to this phenomenon is the universe immunosuppressive microenvironment in tumours or acquired and intrinsic resistance.^5^, ^6^ The tumour microenvironment comprises tumour cells, immune cells, cytokines and stromal cells. Communication and interaction occur between these components. In many tumours, the function of immune cells that kill cancer cells is often compromised, while immune suppressor cells accumulate excessively and further inhibit immune responses, facilitating tumour progression. This indicates that tumour microenvironment dysregulation is pivotal in driving persistent tumour growth, facilitating metastasis, and significantly influencing the response to immunotherapy. Moreover, an increasing body of evidence has demonstrated that abnormal gene expression and the build up of mutations in oncogenes or tumour suppressors significantly influence the surrounding tumour microenvironment and the body's immune responses, promoting the advancement of cancer.^7^, ^8^ Thus, the identification of novel molecular targets that can effectively reverse the immunosuppressive microenvironment and slow down tumour growth is urgently needed.

The dynactin family encompasses a group of proteins associated with the dynactin complex, a crucial factor in cellular processes closely associated with tumour development.^9^, ^10^ Among these, dynactin subunit 2 (DCTN2) have emerged as a potential significant player in tumorigenesis. In colon cancer, DCTN2 binding with ROCK1 stimulates tumour progression by modulating centrosome amplification.^11^ Additionally, DCTN2 functions as a FER substrate, which is essential for the development of tubular recycling domains in early endosomes and subsequent propagation of TNBC cell invasion.^12^ However, the functional contribution and expression pattern are still largely unknown.

In this study, we performed a thorough pan‐cancer analysis to examine the molecular features and prognostic implications of DCTN2 using data from multiple databases. We carried out comprehensive functional and pathway enrichment analyses to explore the involvement of DCTN2 in tumorigenesis. Additionally, we accessed the correlation between DCTN2 and immune biomarkers, shedding light on their relevance in the context of tumour immunity. Finally, the bioinformatics results were validated using HCC cell lines. This study offers valuable insights into the significance of DCTN2 in diverse types of cancers, highlighting the promising therapeutic potential of targeting DCTN2 in the treatment of HCC.

## 2 MATERIALS AND METHODS

### 2.1 Pan‐cancer expression data and public data acquirement

To facilitate a comprehensive understanding of DCTN2 expression, pan‐cancer expression data of the The Cancer Genome Atlas (TCGA) and/or the Genotype‐Tissue Expression Project (GTEX) project were gathered from the UCSC XENA database (http://xena.ucsc.edu/) or obtained from Tumour‐Infiltrating Immune Cells (TIMER) and Sangerbox database (http://sangerbox.com/Tool).^13^, ^14^ The expression pattern of DCTN2 in different kinds of malignancies was analysed and plotted. Moreover, the cBioPortal database (https://www.cbioportal.org) facilitated the acquisition and graphical depiction of DCTN2 expression in conjunction with somatic mutations in various cancer types.^15^ The Human Protein Atlas (HPA) database^16^ was applied to obtain DCTN2 protein level in tumour tissues and normal ones of HCC. Additional expression data for DCTN2 were obtained from the Gene Expression Omnibus (GEO) database (https://www.ncbi.nlm.nih.gov/geo/) and used for comparative analysis to verify the variations of DCTN2 expression in HCC and adjacent normal tissues.

### 2.2 Prognostic analysis in pan‐cancer

In order to explore the potential prognostic significance of DCTN2 in a broad range of cancer types, the Kaplan–Meier analyses and univariate Cox regression were conducted to determine the prognostic value of DCTN2 on overall survival in cancers after performing data quality control, including the exclusion of patients with incomplete survival information. *p* < 0.05 were considered as statistical significance.

### 2.3 Functional enrichment of DCTN2 in pan‐cancer

After obtaining the Pan‐cancer expression data from the UCSC XENA database, we conducted differential gene analysis to determine genes expressed differentially with respect to DCTN2 expression via R package “limma.”^17^ We selected the top 30 and bottom 30 percent samples based on DCTN2 expression in each tumour type. We performed pan‐cancer GSEA to assess the enrichment of biological pathways and gene sets in MSigDB database^18^ that associated with DCTN2 expression with the R package “clusterProfiler.”^19^ This analysis helped identify functional associations between DCTN2 and specific pathways or biological processes. For specific pathways, we calculated the pan‐cancer *z*‐scores of each sample and the relationship between these pathways and DCTN2 expression.

### 2.4 The association of DCTN2 with pan‐cancer immune cell infiltration

To assess the association between DCTN2 and the pan‐cancer immune context, we employed four computational algorithms: QUANTISEQ, EPIC, MCP‐counter and TIMER via the Sangerbox database. Additionally, we investigated the potential impact of DCTN2 on immune regulation by examining its association with immune suppression genes and IPS.

### 2.5 Cell culture and lentivirus infection

HCC cell lines (MHCC97H, Huh‐7, HCCLM3 and HepG2) were obtained from the Cell resource center, Shanghai institute of life sciences, Chinese academy of sciences (Shanghai, China) and cultured in Dulbecco's modified Eagle's medium (BasalMedia, Shanghai, China) supplemented with 10% fetal bovine serum (FBS, Biological Industries) and 1% penicillin–streptomycin (NCM Biotech, Jiangsu, China). Cells were incubated in a humidified incubator.

For stable DCTN2 knockdown cell construction, sh‐DCTN2 or a negative control (sh‐NC) lentivirus (GeneChem, Shanghai, China) was used to infect the cells following the manufacturer. After 48–72 h, puromycin (2 μg/mL, Beyotime, Shanghai, China) was added into the culture medium to screen out the stably infected cells. DCTN2 inhibition efficacy was confirmed using qPCR and western blot analyses.

### 2.6 Total RNA extraction and real‐time qPCR

Total RNA was extracted from cells using TRIzol reagent.cDNA was obtained, and qPCR was performed using specific primers for DCTN2 and reference genes using SYBR green reagent (Vazyme, Jiangsu, China). The qPCR Primers sequence are as followings. DCTN2‐Forward: 5′‐CCCAGATAGCAGCCTTGTCACT‐3′, Reverse: 5′‐TCCAGCTCTGTCAGGCGCTTTT‐3′; GAPDH‐Forward: 5′‐GTCTCCTCTGACTTCAACAGCG‐3′, Reverse: 5′‐ ACCACCCTGTTGCTGTAGCCAA‐3′.

### 2.7 Cell proliferation assay

Cell Counting Kit‐8 (CCK‐8, NCM Biotech, Jiangsu, China) assay was used to assess the cell proliferation capability. Briefly, 3000 cells were grown in a 96‐well plate. On days 1, 2, 3 and 4, the cell viability and proliferation was determined by absorbance value at 450 nm.

### 2.8 Cell migration and invasion assays

Cell migration and invasion were analysed using scratch wound healing and transwell invasion assays with Matrigel (R&D Systems, USA), respectively. For migration, cells seeded in the 6‐well plates were gently scraped using a 200 μL pipette tip. Then PBS was used to remove the debris or cell fragments, taking photos under a microscope at 0 and 36 h, respectively. To assess invasion ability, a Transwell chamber (8 μm pore size, Corning Costar, USA) was used after incubating with Matrigel at 37°C for 15 min and then inoculated with 200 μL of serum‐free cell suspension containing 50,000 cells. In the lower chamber, 600 μL of culture medium supplemented with 20% fetal bovine serum was added. Following a 24‐h incubation, 4% paraformaldehyde and 0.5% crystal violet dye were used to fix and stain.

### 2.9 Western blot analysis

Cellular proteins were collected using cell lysis supplemented with PMSF. BCA protein quantification was performed, and the samples were denatured by boiling in a water bath. Subsequently, the denatured protein samples were stored at −80°C.The equal amounts of proteins were added to a suitable 10% SDS‐PAGE grid system and then electrolyzed and transferred to a PVDF membrane. After blocking with 5% BSA, primary antibodies and corresponding secondary antibodies were incubated, respectively. The antibodies used are as followings. Rabbit polyclonal to DCTN2 (bs‐7836R, Bioss Antibodies), Rabbit polyclonal to AKT (10176‐2‐AP, Proteintech), Mousemonoclonal to p‐AKT (66444‐1‐Ig, Proteintech), Rabbit polyclonal to mTOR (28273‐1‐AP, Proteintech), Rabbit polyclonal to p‐mTOR (80596‐1‐RR, Proteintech), Mousemonoclonal to GAPDH (60004‐1‐Ig, Proteintech).

### 2.10 Drug treatment

AKT agonist SC‐79 (4 μg/mL, abs810216, absin, Shanghai, China) was added into the culture medium of HCC cells. All cells were collected and counted for functional experiments or lysed in RIPA buffer after 48 h treatment.

### 2.11 Statistical analysis

Data visualization was conducted using R and GraphPad Prism 6.To compare gene expression differences, the Student's *t*‐test was applied. Spearman's correlation analysis was used to analyse the association between two groups. Survival characteristics were assessed using the Kaplan–Meier method and/or Cox regression analysis.

## 3 RESULTS

### 3.1 DCTN2 expression pattern in pan‐cancer

**FIGURE 1:**
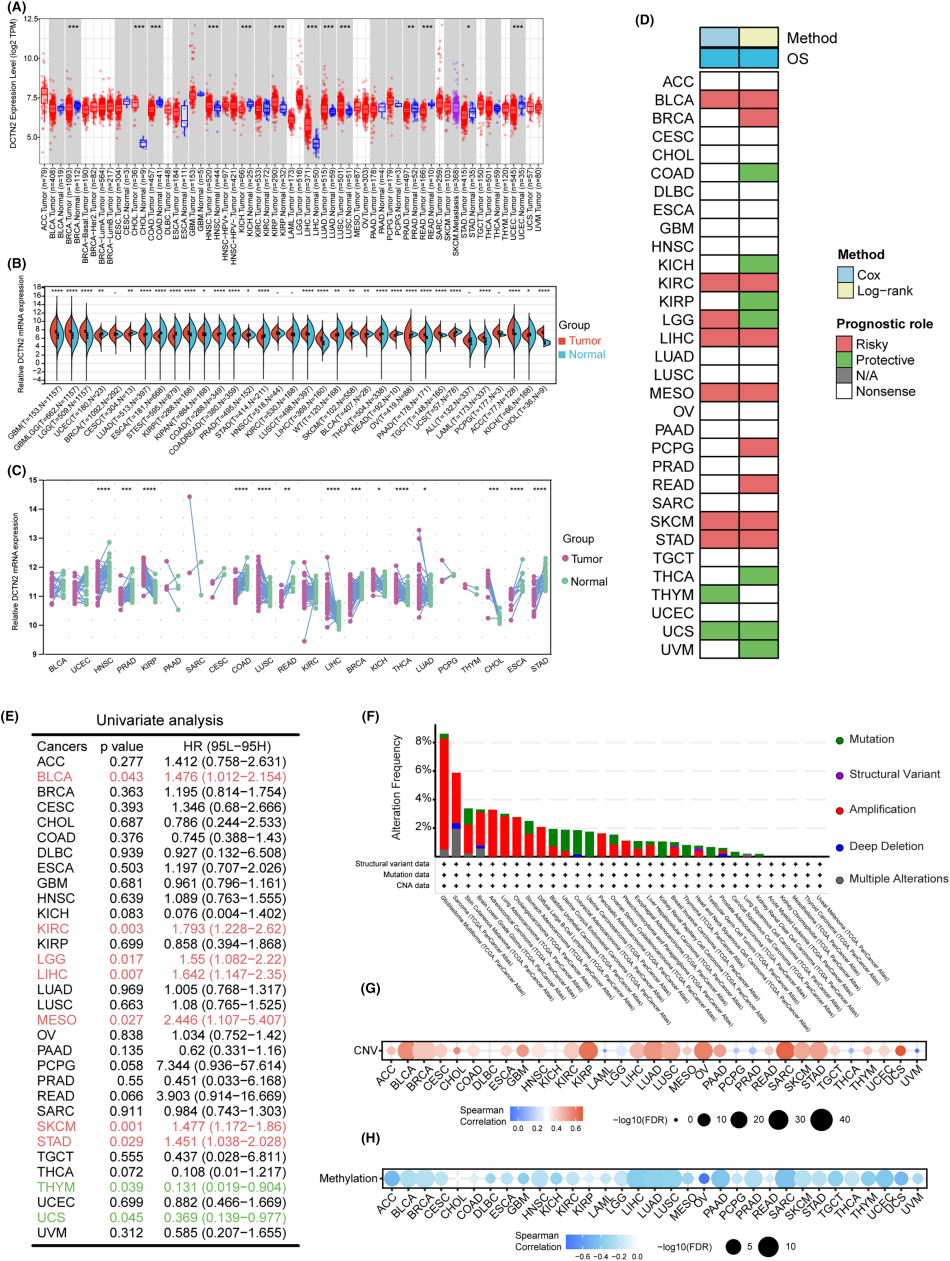
DCTN2 expression pattern in human tumour tissues and normal tissues. (A) Boxplot showing the mRNA expression levels of DCTN2 in normal and cancer tissues using data from the TIMER database based on TCGA data. (B) Violin plot illustrating the mRNA expression levels of DCTN2 in normal and tumour tissues using data from the Sangerbox database based on TCGA&GTEX data. (C) DCTN2 mRNA expression in paired tumour and adjacent non‐tumour tissues based on TCGA data. (D) Heatmap showing the correlation between DCTN2 expression levels and overall survival (OS). (E) Univariate analysis demonstrating the prognostic value of DCTN2 in various tumours. (F) The somatic mutation of DCTN2 in various type of cancers. (G, H) Dotplot showing the correlation of DCTN2 with CNV or DNA methylation. *p < 0.05, **p < 0.01, ***p < 0.001, ****p < 0.0001.

We first utilized Tumour Immune Estimation Resource (TIMER) online analysis database to delve the expression status of DCTN2 in various cancers. The results indicated that DCTN2 expression was dysregulated in most tumours, with elevated expression in many type of cancers, such as HCC, glioblastoma (GBM), pancreatic cancer and decreased expression in other cancers, such as breast cancer, colocteral cancer and ovarian cancer (Figure 1A). We then expanded the analysis using the GTEX database to include normal samples via the Sangerbox online database, and the results were consistent (Figure 1B). Moreover, paired analysis further revealed significantly higher expression of DCTN2 in many tumour tissues (Figure 1C). Next, we explored its prognostic value in various tumours. The results of the survival analysis demonstrated that increased expression of DCTN2 was linked to reduced overall survival rates across multiple cancer types, such as bladder cancer, kidney renal clear cell carcinoma and liver hepatocellular carcinoma (LIHC) (Figure 1D,E). Subsequently, we investigated the epigenetic regulatory mechanisms underlying the dysregulation of DCTN2. Somatic mutation analysis revealed that the most common type of mutation was amplification, including various tumour types, with a significant presence in glioblastomas (Figure 1F). Additionally, we unveiled a robust positive correlation between DCTN2 expression and copy number alterations in most tumour specimens, while a noteworthy inverse relationship was detected between DCTN2 expression and gene methylation levels (Figure 1G,H).These findings suggest that DCTN2 is dysregulated in multiple cancers, indicating its potential role in tumour development.

### 3.2 Enrichment analysis of DCTN2 in pan‐cancer

**FIGURE 2:**
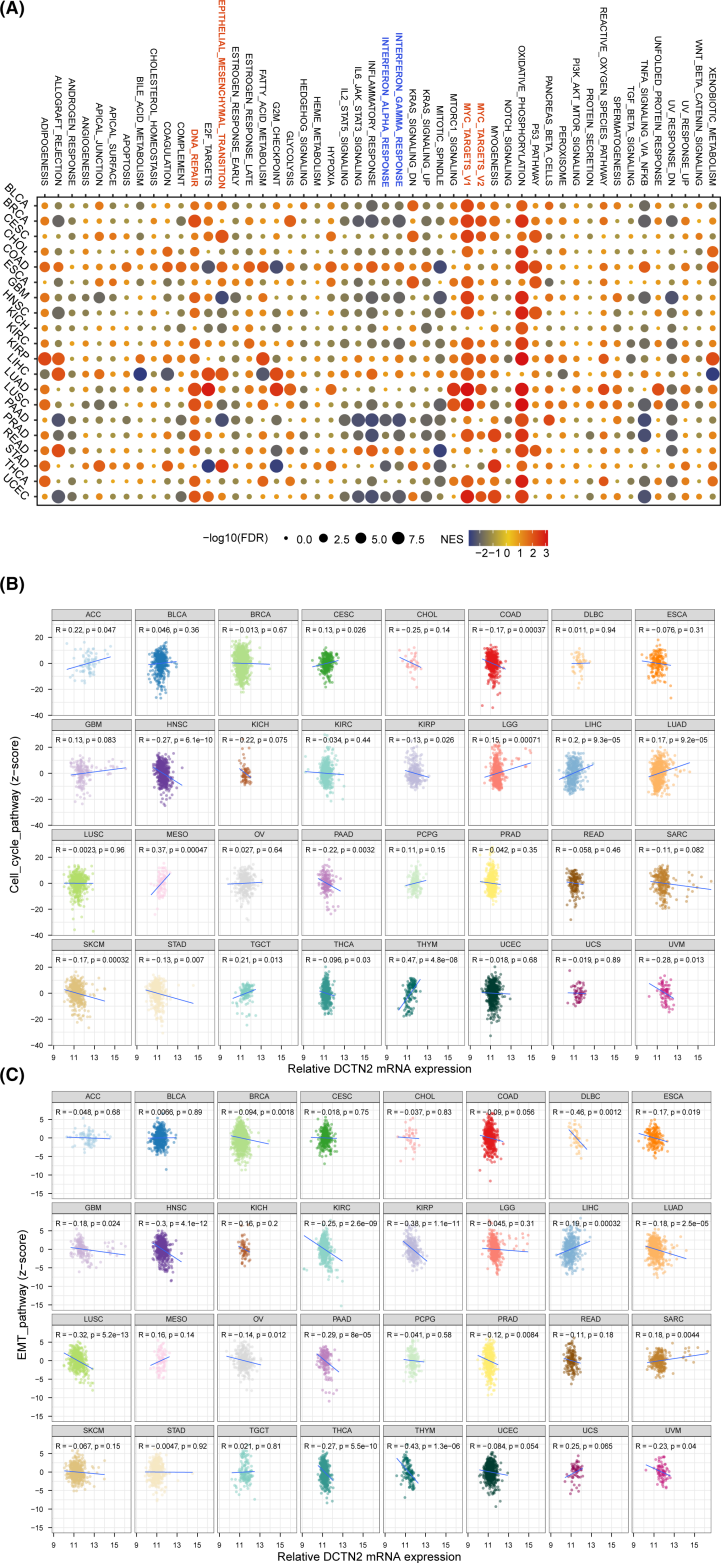
Potential function analysis of DCTN2 in human cancers. (A) Bubble plot drawing the results of GSEA between DCTN2‐high and ‐low tumour patients analysed by HALLMARKER gene set. (B, C) Scatter plots showing the correlation between DCTN2 expression and cell cycle pathway score or EMT pathway score.

To access the function patterns of DCTN2 in various cancers, pan‐cancer expression profile data were retrieved from the UCSC XENA database. The top and bottom 30 percent samples, based on DCTN2 expression, were selected for each tumour type, and differential gene analysis was conducted using the limma package. Pan‐cancer gene set enrichment analysis (GSEA) was then conducted, revealing a significant positive association between DCTN2 and oncogenic pathways, such as epithelial‐mesenchymal transition (EMT), MYC_TARGETS and DNA_REPAIR but a negative correlation with INTERFERON_ALPHA_RESPONSE and INTERFERON_GAMMA_RESPONSE pathways (Figure 2A). The correlation between DCTN2 and these pathways was explored using the *z*‐score algorithm in ssGSEA. The analysis demonstrated a positive correlation between DCTN2 expression and cell cycle and EMT pathways in multiple tumours (Figure 2B,C). These findings suggest that high DCTN2 expression is closely linked to various pro‐carcinogenic pathways, indicating its potential role in promoting tumour progression.

### 3.3 The association of DCTN2 with immune infiltration

**FIGURE 3:**
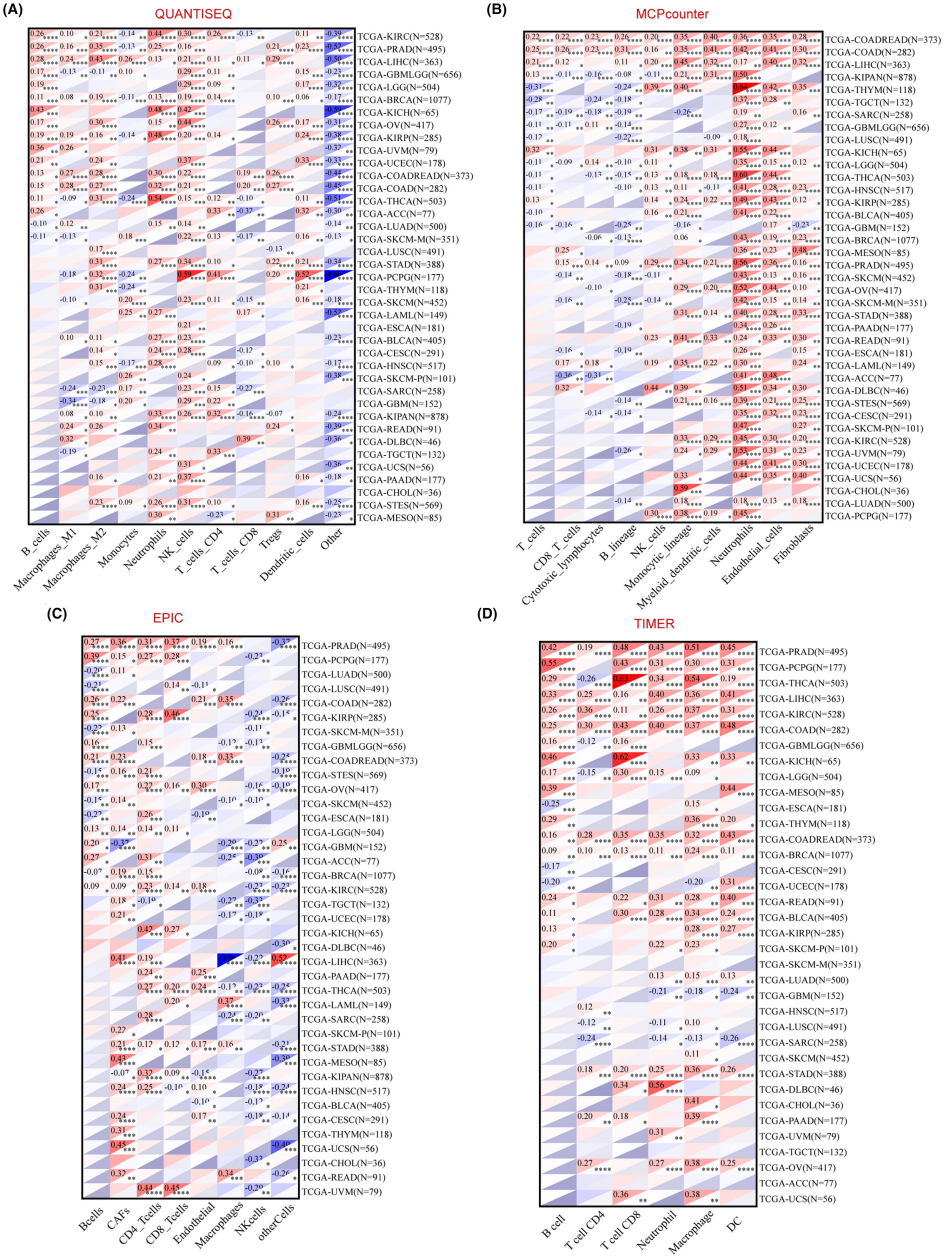
Correlation analysis between DCTN2 expression and immune cell infiltration. (A–D) heatmaps display the correlation between DCTN2 expressions and the degree of infiltration of various immune cells by four different kinds of analysis methods.*p < 0.05, **p < 0.01, ***p < 0.001, ****p < 0.0001.

To investigate the relationship between DCTN2 and immunological responses, we used four established algorithms (Quantification of Tumour Immune Contexture from human RNA‐seq data [QUANTISEQ], Microenvironment Cell Populations counter [MCPcounter], Estimating the Proportion of Immune and Cancer cells [EPIC], and TIMER) to evaluate the relationship of pan‐cancer immune cell infiltration with DCTN2 expression. DCTN2 significantly correlated with neutrophils, macrophages, and cancer‐associated fibroblasts (CAFs) in most types of cancer, including liver cancer, thyroid cancer, ovarian cancer, and stomach carcinoma (Figure 3A–D). These cells have been demonstrated to promote immunosuppressive microenvironment formation and tumour progression, indicating that DCTN2 plays an immunosuppressive role in various cancers.

**FIGURE 4:**
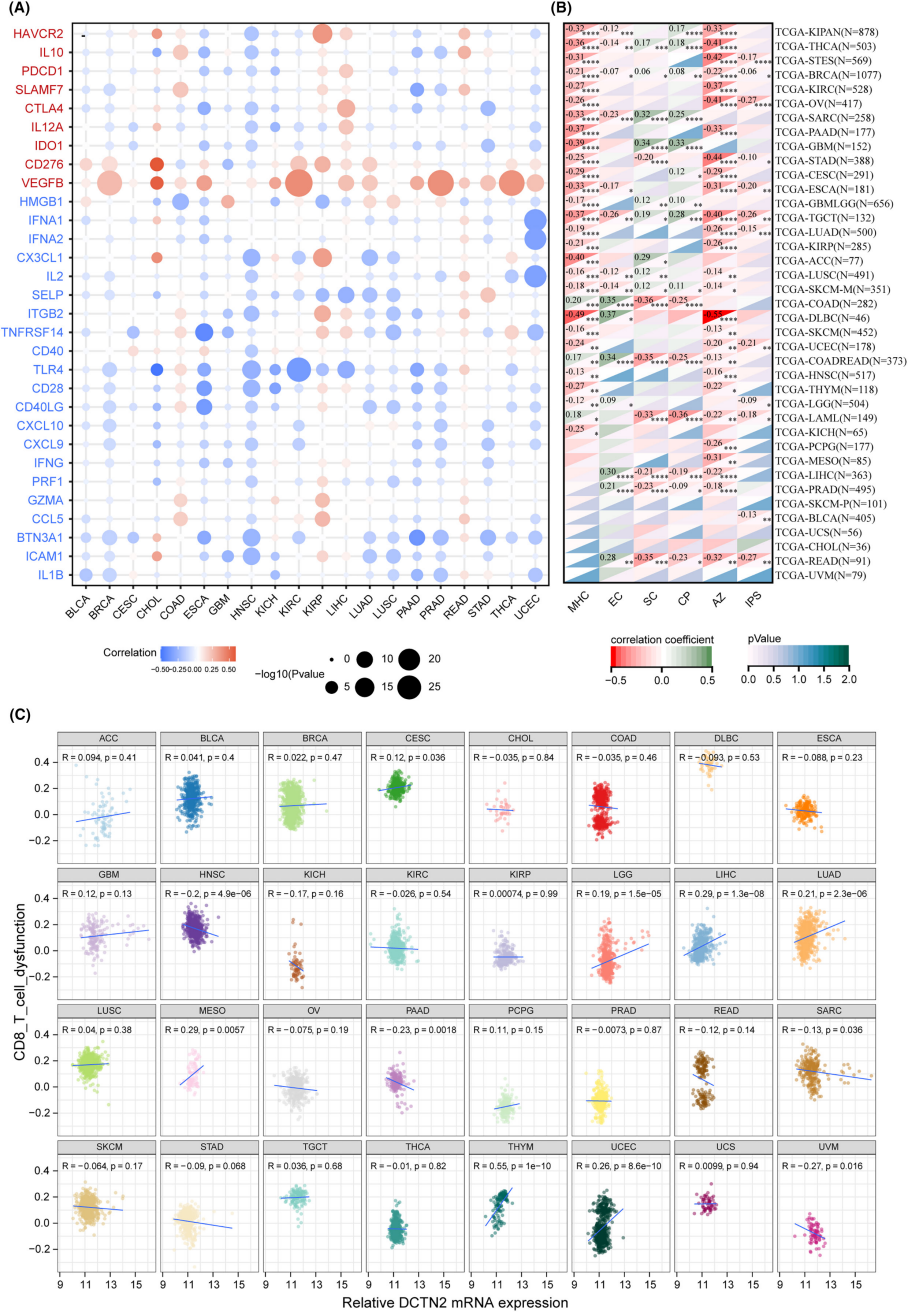
Correlation analysis between DCTN2 expression and immune checkpoints. (A) Dotplot displays the association between DCTN2 and immunoregulators. Gene names in red represent inhibitors and those in blue indicate stimulators (B) Heatmap showing the correlation of DCTN2 with MHC score and IPS score, etc. (C) Scatter plots showing the correlation between DCTN2 expression and CD8 T cell dysfunction score.*p < 0.05, **p < 0.01, ***p < 0.001, ****p < 0.0001.

Furthermore, we analysed the relationship between DCTN2 and immune regulatory genes. The results indicated a notable association of elevated DCTN2 expression with immune inhibitory molecules as well as a negative correlation with the immunophenoscore (IPS) (a positive indicator of immunotherapy sensitivity) (Figure 4A,B).^20^ Additionally, a correlation analysis of CD8^+^ T cell function demonstrated that higher DCTN2 expression was associated with impaired CD8^+^ T cell function (Figure 4C). Our findings demonstrate that high DCTN2 expression correlates with immune inhibitory molecules and compromises CD8^+^ T cell function. Further exploration is warranted to understand the complex interplay between DCTN2 and the immune microenvironment in cancer.

### 3.4 DCTN2 is upregulated in HCC tissue and correlated with poor prognosis

The above findings demonstrated that DCTN2 was dramatically overexpressed in HCC, and correlated with poorer overall survival in patients with HCC. These correlations were also significant with the IPS, immune‐inhibitory genes, and cell cycle scores. Therefore, we investigated the possible biological functions and mechanisms of action of DCTN2 in HCC.

**FIGURE 5:**
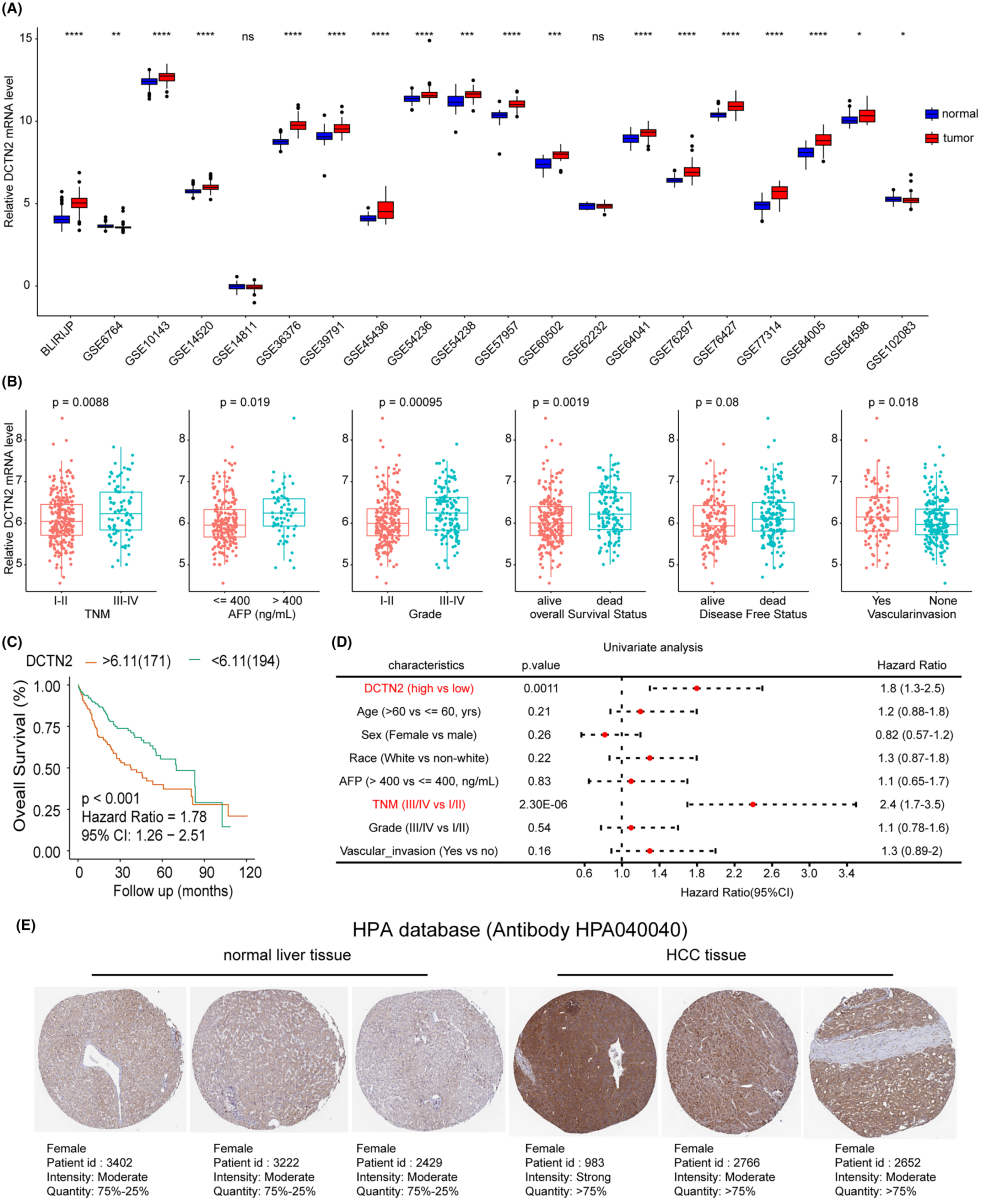
DCTN2 is highly expressed in HCC and correlates with poor prognosis. (A) Boxplot showing the expression of DCTN2 in tumour and normal tissues in various GEO HCC datasets. (B) Higher DCTN2 expression was observed in patients with late TNM stage, higher AFP levels, poor differentiation grade and vascular invasion. (C, D) Kaplan–Meier and univariate analysis showing high DCTN2 associated with poor prognosis. (E) DCTN2 protein was higher in HCC tissues than normal tissues from HPA database.*p < 0.05, **p < 0.01, ***p < 0.001, ****p < 0.0001, NS: not significant.

We selected and downloaded several other HCC datasets from a public database to further determine the expression level of DCTN2. As expected, much higher expression of DCTN2 was observed in HCC tissues (Figure 5A). Additionally, high DCTN2 levels were observed in patients with advanced TNM stage, high AFP levels, vascular invasion, and differentiation grade (Figure 5B). Further survival analysis showed that HCC patients with high DCTN2 expression had poorer clinical outcomes (Figure 5C). Subsequent univariate and multivariate analyses identified DCTN2 expression and TNM stage as independent prognostic factors in HCC patients (Figure 5D; Table 1). Additionally, the HPA database^16^ also revealed higher DCTN2 protein expression in HCC tissues (Figure 5E). Our findings strongly suggest the crucial involvement of DCTN2 in HCC development and progression.

**TABLE 1:** Multivariate analyses of DCTN2 expression in HCC patients from TCGA‐LIHC cohort.

Characteristics	Multivariate analyses	
	Hazard ratio	*p*‐value
DCTN2 expression		
High versus low	1.7 (1.2–2.5)	0.0043**
TNM stage		
III–IV versus I–II	2.4 (1.6–3.5)	<0.001***

*Note*:**p < 0.01, ***p < 0.001.

Abbreviation: TNM: tumour‐node‐metastasis.

### 3.5 Effect of DCTN2 on HCC cell behaviour

**FIGURE 6:**
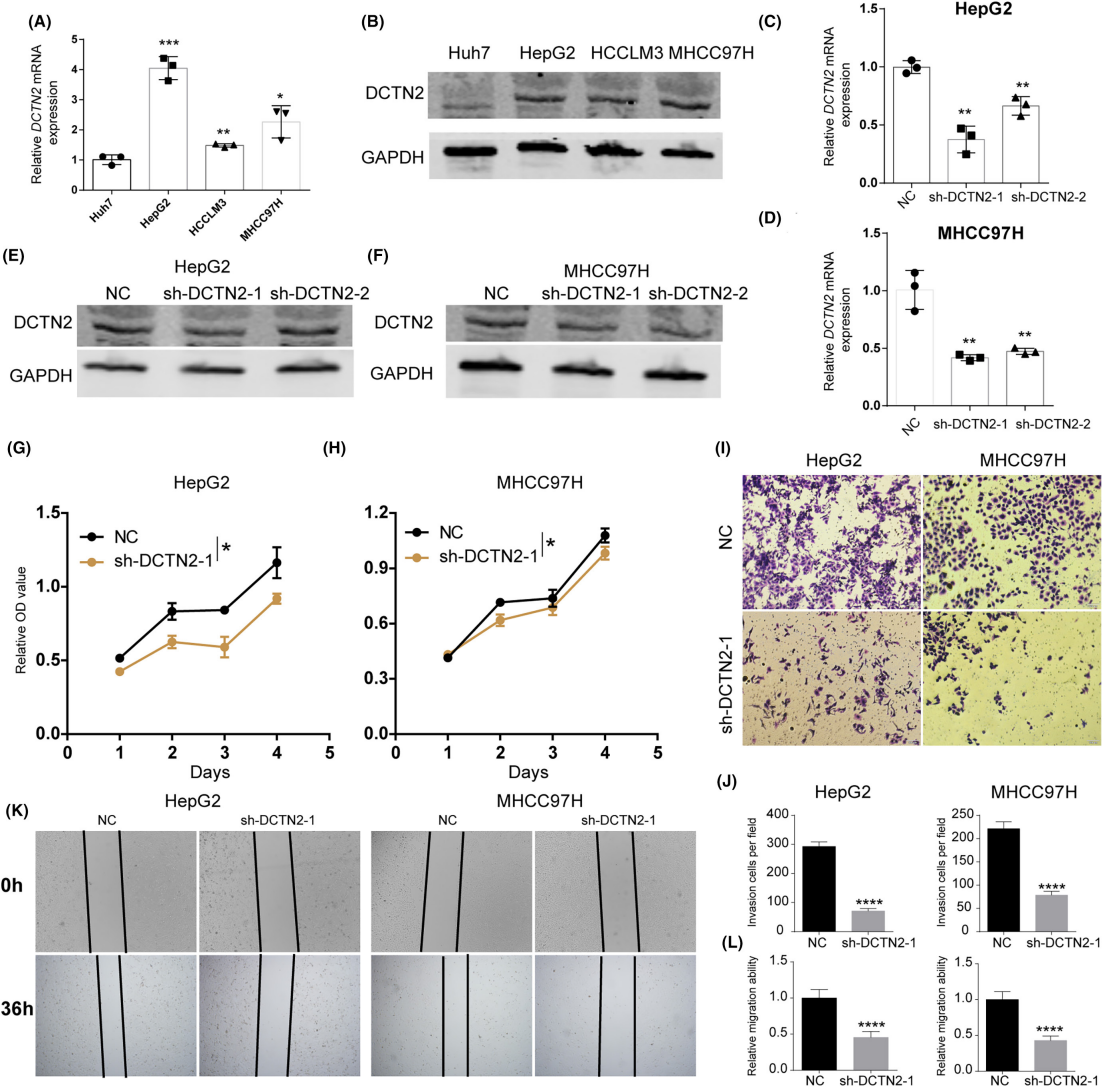
DCTN2 knockdown inhibits HCC cell proliferation and metastasis. (A, B) qPCR and western blot showing the expression of DCTN2 in several HCC cell lines. (C–F) qPCR and western blot confirmed the konckdown efficiency of DCTN2 in HepG2 and MHCC97H cell lines. (G, H) CCK‐8 assay demonstrated that DCTN2 silence repressed HCC cell proliferation. (I–L) Transwell invasion and wound healing assays showed DCTN2 knockdown inhibited the metastasis capacity of HCC cells. *p < 0.05, **p < 0.01, ***p < 0.001, ****p < 0.0001.

To determine the expression levels of DCTN2 in HCC cell lines, quantitative PCR (qPCR) and western blot analyses were performed. Our results demonstrated significant upregulation of DCTN2 expression in multiple HCC cell lines (Figure 6A,B). According to their expression levels, we selected the MHCC97H and HepG2 cell lines for subsequent experiments. Next, stable knockdown of DCTN2 was achieved in these two HCC cell lines, and the knockdown efficiency was validated by qPCR and western blot analysis (Figure 6C–F). Functional assays revealed a notable reduction in the proliferative capacity of HCC cells following DCTN2 knockdown (Figure 6G, H). The results obtained from the wound healing and transwell invasion assays exhibited a significant decrease in the migratory and invasive capabilities of the DCTN2 knockdown group, in comparison to the control group (Figure 6I–L). These observations strongly suggest that the suppression of DCTN2 can effectively inhibit the aggressive behaviour of HCC cells.

### 3.6 Enrichment analysis of DCTN2 in HCC

**FIGURE 7:**
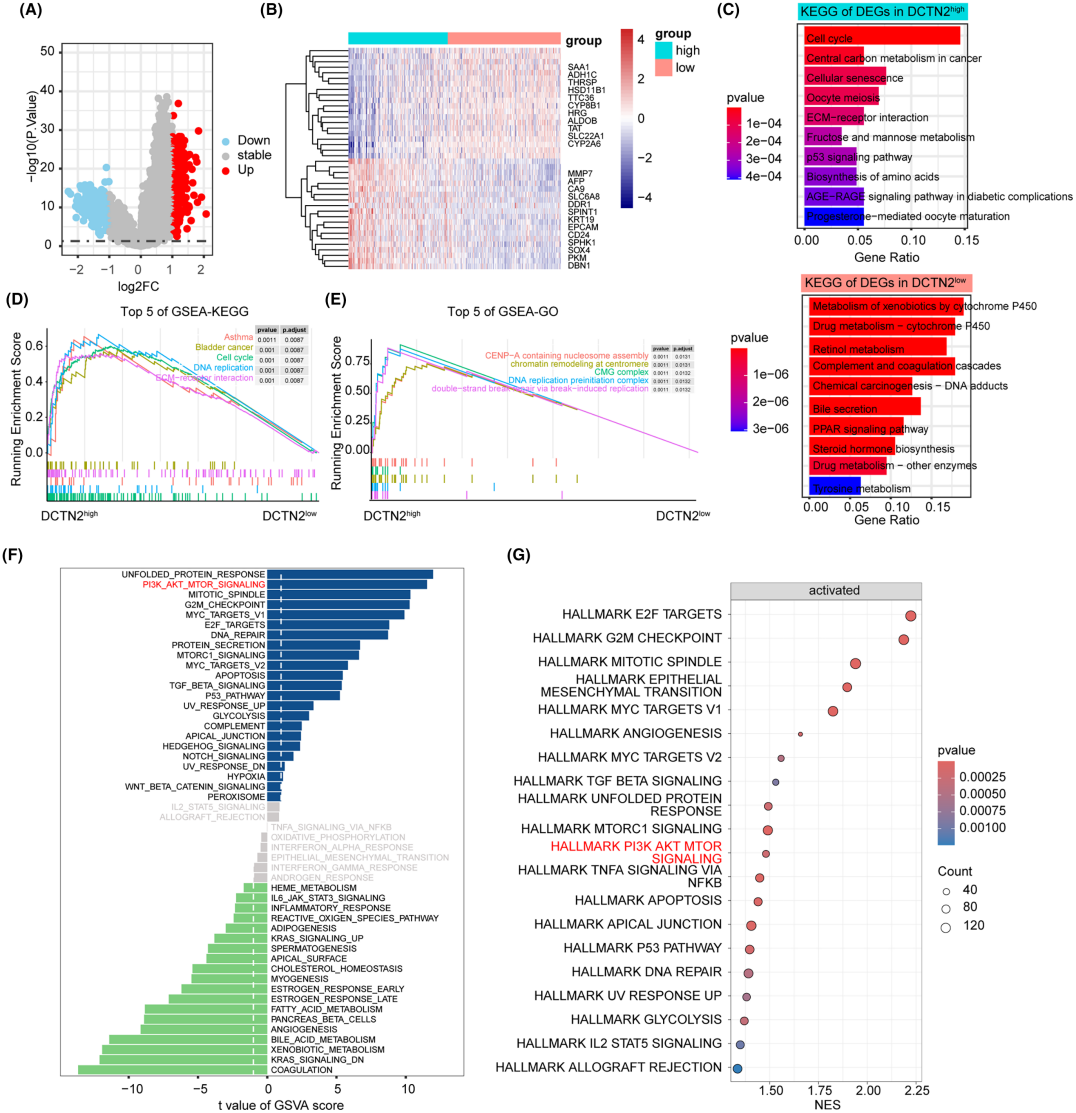
Potential function analysis of DCTN2 in HCC. (A) Volcano plot showing the differential expressed genes (DEGs) in DCTN2 high and low groups. (B) Heatmap displayed the top DEGs in DCTN2 high or low group. (C) Barplot illustrating pathways enrichment in DCTN2 high or low group. (D, E) The top 5 pathways enriched by GSEA‐KEGG and GSEA‐GO analysis. (F, G) Hallmarker analysis by GSVA and GSEA showing AKT pathway was significantly enriched in DCTN2 high group.

Given the inhibitory effects of DCTN2 on HCC cell behaviour, we explored the underlying molecular mechanisms. We categorized the patients with HCC into the DCTN2 high and the DCTN2 low group based on the best cut‐off value of DCTN2 in the survival analysis. We identified a set of differentially expressed genes, with the majority of genes in the DCTN2 high group being associated with cell proliferation and stemness (Figure 7A,B). To gain further insights into their functional implications, we conducted gene ontology (GO) and Kyoto Encyclopedia of Genes and Genomes (KEGG) analyses. The results revealed that genes in the DCTN2 high group were enriched in “cell cycle”, “cellular senescence” and “ECM‐receptor interaction” (Figure 7C). Similar results were observed in GSEA‐KEGG and GSEA‐GO analyses (Figure 7D,E). Furthermore, GSVA and GSEA‐HALLMARKER analyses revealed that DCTN2 was positively associated with tumour‐promoting pathways such as the PI3K/AKT/mTOR pathway, MYC_targets, and E2F_targets, indicating that DCTN2 was significantly related to various tumour‐related pathways (Figure 7F,G).

### 3.7 DCTN2‐mediated modulation of the AKT pathway

**FIGURE 8:**
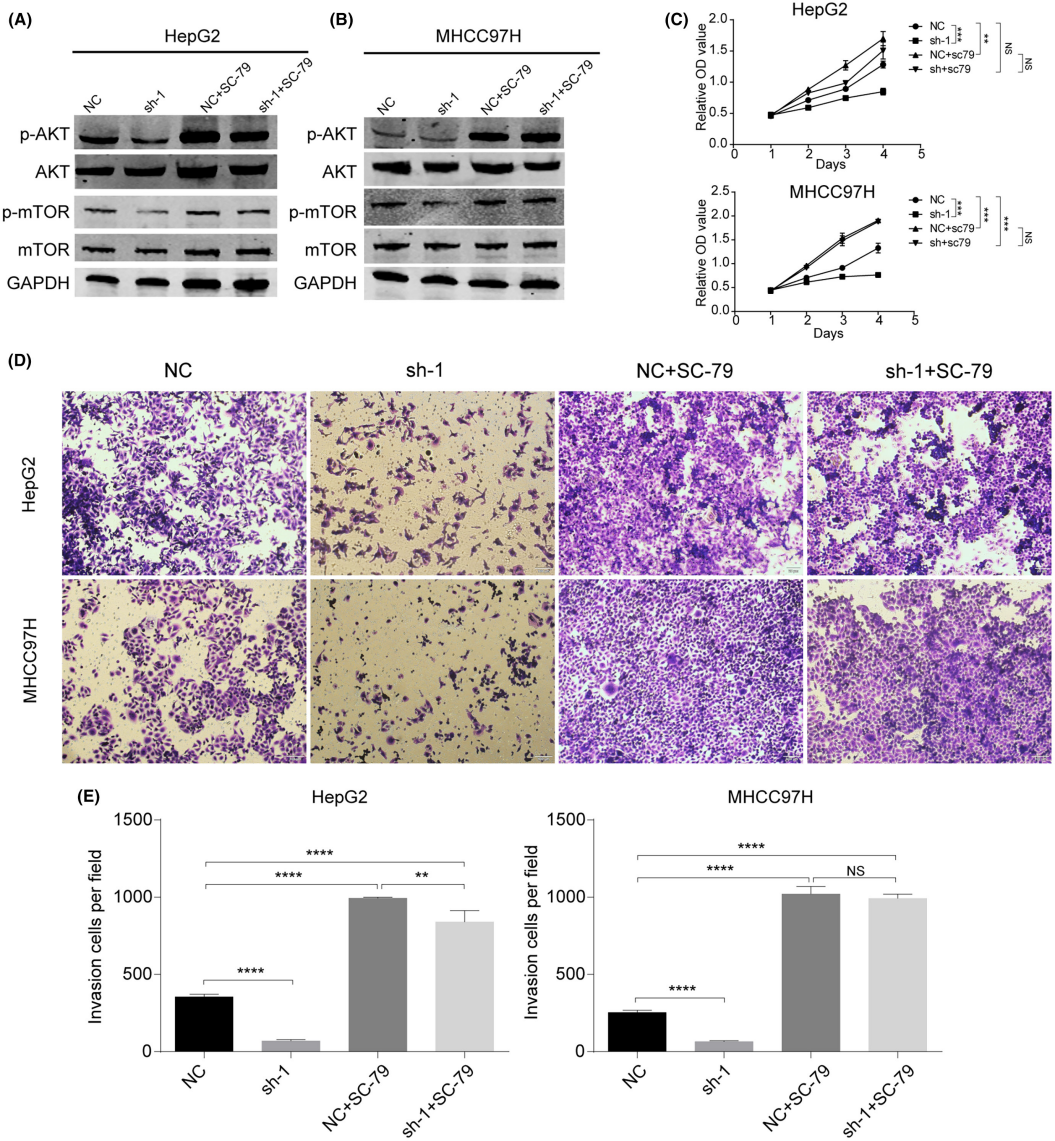
DCTN2 accelerated HCC progression by activating AKT pathway. (A, B) Knockdown DCTN2 suppressed AKT phosphorylation, while this was partial reversed by SC‐79, an AKT pathway activator. (C) CCK‐8 assay demonstrated that the inhibitory ability to HCC cells proliferation after DCTN2 silence was counteracted by SC‐79. (D, E) Transwell invasion assay demonstrated that the inhibitory ability to HCC cells metastasis after DCTN2 silence was counteracted by SC‐79. **p < 0.01, ***p < 0.001, ****p < 0.0001; NS, not significant.

We validated the involvement of the AKT pathway in DCTN2‐mediated tumour progression through western blot analysis, which revealed that DCTN2 inhibition led to a substantial decrease in AKT phosphorylation levels. Additionally, the downstream targets of the AKT pathway, such as mTOR, exhibited reduced phosphorylation upon DCTN2 knockdown (Figure 8A,B). Subsequently, the rescue experiments were performed. We treated HCC cells with an AKT agonist, SC‐79. AKT activation partially rescues the inhibitory effects of DCTN2 silencing on cell proliferation and invasion, suggesting that the AKT pathway plays a role in mediating the tumour‐promoting effects of DCTN2 (Figure 8C–E). These findings indicate that the suppression of DCTN2 can partially inhibit the AKT pathway, leading to the attenuation of malignant biological behaviours in HCC cells.

## 4 DISCUSSION

Although several studies have suggested that abnormal expression of DCTN2 may be associated with tumorigenesis and tumour progression the exact role of DCTN2 in various cancers is not yet fully understood, and its potential connection with antitumor immune responses warrants further investigation. In this study, we examined DCTN2 expression in a pan‐cancer setting. DCTN2 exhibited a predominantly high expression profile in all tumour samples from the TCGA database, which alluded to its potential role as an oncogenic driver for tumorigenesis and tumour progression. The survival analysis indicated that DCTN2 overexpression had a negative prognostic impact on the clinical outcomes of patients with cancer. These findings underscore the potential significance of DCTN2 as a prognostic biomarker across different types of malignancies.

The pivotal role of the tumour immune microenvironment cannot be overstated. The dynamic interplay between immune cells and the tumour, whether it exhibits an inflamed (hot) or non‐inflamed (cold) phenotype, substantially affects the response to immunotherapeutic modalities.^21^, ^22^, ^23^ However, numerous factors hamper the optimal infiltration of immune cells into the tumour microenvironment in most patients.^24^ Moreover, functional impairment caused by cytotoxic immune cells over time severely compromises their ability to recognize and eliminate neoplastic cells.^25^, ^26^, ^27^ Consequently, identifying key molecular alterations capable of addressing these obstacles and augmenting the efficacy of immunotherapeutic strategies is an urgent research priority.

The outcomes of our scientific inquiry revealed a compelling association between heightened DCTN2 expression and the fostering of an immunosuppressive microenvironment. Notably, DCTN2 is closely related to the infiltration patterns of numerous immune cell subsets in various malignancies, with a notable positive correlation specifically observed with the infiltrative propensity of CAFs. CAFs are a type of cells found in the tumour microenvironment and originate from normal fibroblasts.^28^, ^29^ They are considered key drivers of tumour‐related inflammation and secrete various cytokines and proteins, such as oncogenic factors, growth factors and extracellular matrix proteins, to influence tumour cell growth and infiltration. Additionally, CAFs can modulate immune responses by interacting with immune cells and regulating immune infiltration and antitumor immune responses in the tumour microenvironment.^30^, ^31^ Furthermore, patients with elevated DCTN2 expression exhibited decreased IPS and increased expression of immune‐inhibitory genes. These findings suggest that DCTN2 might engage in a positive feedback loop with specific immune checkpoint genes, promoting immune evasion by tumour cells. These mechanisms emphasize the potential predictive and therapeutic utility of DCTN2 in precision oncology treatments.

In addition, we explored the expression and potential prognostic significance of DCTN2 in HCC and its functional role in HCC cell behaviour. Our findings revealed that DCTN2 expression was prominently higher in HCC tissues. Cox regression analysis told that it could be an independent risk factor for HCC, consistent with its potential tumour‐promoting role. Functional experiments performed in this study demonstrated that DCTN2 knockdown significantly inhibited HCC cell proliferation, migration and invasion.

To investigate the molecular mechanisms by which DCTN2 exerts the tumour‐promoting effects, we examined the AKT pathway, a well‐known signalling pathway implicated in cancer progression.^32^, ^33^ Aberrant activation of this pathway has been observed in breast cancer, colorectal cancer, lung cancer and HCC and so on.^34^, ^35^, ^36^, ^37^ In each of these malignancies, AKT activation promotes cell survival, proliferation, metastasis and stemness, making it an attractive target for therapeutic intervention. Consistently, our results revealed that DCTN2 inhibition decreased the phosphorylation of AKT and its downstream targets, including mTOR. These findings suggest that DCTN2 modulates the AKT pathway to inhibit HCC cell behaviour. Moreover, rescue experiments using SC79, an AKT agonist,^38^ partially restored the proliferative and migratory abilities of HCC cells by silencing DCTN2, further supporting the involvement of the AKT pathway in DCTN2‐mediated tumour suppression.

Despite the significant insights gained from this study, it had several limitations. First, the bulk of our findings stems from publicly available databases on the basis of bioinformatics analyses and the sample size of the patient cohort was relatively small, and future studies with larger cohorts are warranted to verify the prognostic significance of DCTN2 expression in HCC. Secondly, the function of DCTN2 in HCC has been predominantly through in vitro experiments. Additionally, the specific molecular mechanisms underlying DCTN2 regulation of the AKT pathway require further investigation.

In conclusion, our study showcased that DCTN2 is upregulated in HCC, and associated with poor prognosis. DCTN2 knockdown inhibits HCC cell proliferation, migration, and invasion in vitro. These effects may be mediated through the modulation of the AKT pathway. Our research findings offer valuable understandings into the participation of DCTN2 in HCC progression and propose its promising potential as a therapeutic target for managing HCC. Further examination is necessary to unravel the precise mechanisms underlying DCTN2 manipulation and to translate these discoveries into clinical applications.

## AUTHOR CONTRIBUTIONS

**Shuning Xu:** Conceptualization (equal); formal analysis (equal); methodology (equal); software (equal); writing – original draft (equal). **Jiyuan Xing:** Formal analysis (equal); methodology (equal); writing – original draft (equal). **Ke Li:** Formal analysis (equal); methodology (equal). **Lei Qiao:** Methodology (equal); resources (equal). **Cheng Zhang:** Methodology (equal); resources (equal). **Yulin Ren:** Methodology (equal); resources (equal). **Ying Liu:** Conceptualization (equal); data curation (equal); supervision (equal).

## ACKNOWLEGEMENTS

We appreciate the contributions from the TCGA and GEO databases.

## FUNDING INFORMATION

No Funding.

## CONFLICT OF INTEREST STATEMENT

The authors declare that there is no conflict of interests.

## Data Availability

The datasets used and analyzed during the current study are available from the corresponding author on reasonable request.
